# Hippocampal expression of the cannabinoid receptor type 1 in canine epilepsy

**DOI:** 10.1038/s41598-023-29868-3

**Published:** 2023-02-23

**Authors:** D. Kostic, M. Nowakowska, J. Freundt Revilla, F. Attig, K. Rohn, F. Gualtieri, W. Baumgärtner, H. Potschka, A. Tipold

**Affiliations:** 1 Department of Small Animal Medicine and Surgery, University of Veterinary Medicine Hannover Foundation, Hanover, Germany; 2 Institute of Pharmacology, Toxicology and Pharmacy, Ludwig-Maximilians-University Munich, Munich, Germany; 3 Department of Pathology, University of Veterinary Medicine Hannover Foundation, Hanover, Germany; 4 Institute of Biometry, Epidemiology, and Information Processing, University of Veterinary Medicine Hannover Foundation, Hanover, Germany

**Keywords:** Epilepsy, Target identification

## Abstract

Canine drug-resistant epilepsy is a prevailing issue in veterinary neurology. Alternative or additional treatment with cannabinoids is showing promising results in seizure management. A crucial component of the endocannabinoid system, cannabinoid receptor type 1 (CB1R), is heavily involved in the control of neurotransmitter release. Knowledge of its distribution in the epileptic brain would serve a better understanding of disease pathology and application of cannabinoids in dogs with epilepsy. CB1R distribution was assessed in sub-regions of hippocampus of dogs with idiopathic epilepsy, structural epilepsy and without cerebral pathology. In dogs with idiopathic epilepsy, significantly decreased CB1R expression compared to control animals was observed in CA1. In dogs with structural epilepsy, a significant increase in CB1R signal intensity in comparison to controls was observed. CB1R expression was higher in the structural group as compared to the idiopathic. Double immunofluorescence showed co-localization between CB1R and an astrocytic marker in about 50% of cells, regardless of the diagnosis. In summary, CB1R expression in canine hippocampus undergoes modification by the epileptic process and the direction of this change depends on the etiology of the disease. The distinct disease-associated CB1R expression needs to be considered in new treatment development for dogs with epilepsy.

## Introduction

Epilepsy is the most common chronic neurological disease with a lifetime prevalence of 0.76% in the human population^1^ and 0.6–0.75% in the total canine population^2^. However, some purebred dog lines show a predisposition to develop epilepsy, which results in higher prevalence ranging from 3 to even 33% in some breeds^2,3^, posing this disorder as one of the most relevant for veterinary neurology. In addition, high occurrence of epilepsy resistant to treatment with the most commonly used drugs has to be considered, which is estimated to appear in 20–30% of dogs with epilepsy^4^. Therefore, it is crucial to develop and validate alternative therapeutic approaches.

Based on therapeutic success in humans, application of hemp-based medicines in dogs started to increase rapidly^5^. Delta(9)-tetrahydrocannabinolic acid (Δ9-THC) is a phytocannabinoid present in a hemp plant, which was reported to exert proconvulsant^6,7^, but also putative anticonvulsant effects^8,9^. These effects of Δ9-THC are mediated by its action as a partial and/or full agonist of cannabinoid receptor type 1 (CB1R)^10,11^, an important component of the endocannabinoid system (ECS). ECS curbs neuronal excitability through retrograde CB1R signaling, which responds to endocannabinoids synthesized in the postsynaptic terminal in a response to increasing intracellular Ca^2+^ levels during action potential^12^. CB1R activation on presynaptic terminals suppresses neurotransmitter release into synaptic cleft predominantly by inhibiting voltage-gated Ca^2+^ channels^13^. Thus, hypofunction of any element of the ECS could lead to neuronal hyperexcitability, which may manifest as epileptic seizures.

Studies with mice provided evidence of CB1R as a potential target to stop prolonged seizure activity^14^, delay seizure generalization^15^ and shorten duration of status epilepticus^16^. However, modification of target expression could influence therapeutic responses. Thus, it is of particular interest to test whether disease-associated regulation of CB1R needs to be considered. Several studies aimed to describe its redistribution in regions particularly relevant for seizure generation and epileptogenesis, such as hippocampal formation. Analysis of tissue from human patients with temporal lobe epilepsy indicated that while total levels of hippocampal expression of CB1R seem to be decreased^17^ there is a selective increase on inhibitory neurons^18^. In rat models of chemically induced epileptogenesis, CB1R expression was enhanced in hippocampus both in the early post-insult phase^19^ and the chronic phase with recurrent seizures^18^, indicating the influence of disease development on receptor expression. In mice, severe, frequent seizures were associated with a larger decrease of hippocampal CB1R expression as compared to animals developing milder, less frequent seizures^20^. Thus, dynamics of CB1R expression is closely bound to the course of epilepsy. Their description in canine patients would bring additional insight into disease pathology and therapeutic application of cannabinoids. It is noteworthy to mention that canine epilepsy is typically classified into two types according to the etiology: idiopathic (determined genetically or diagnosed by exclusion of other causes) and structural (originating from forebrain disorders) epilepsy^21^. Even though the two types could be similar in their clinical manifestations, their histopathology and pathophysiology vary and might influence the distribution of CB1R differently. Taking in consideration all of above aspects, the aim of this study was to investigate CB1R expression patterns in the hippocampus of dogs with epilepsy and to qualitatively and quantitatively analyze it with regard to different etiologies of the disease and to test the hypothesis that CB1R expression differs between these two etiologies.

## Results

**Figure 1:**
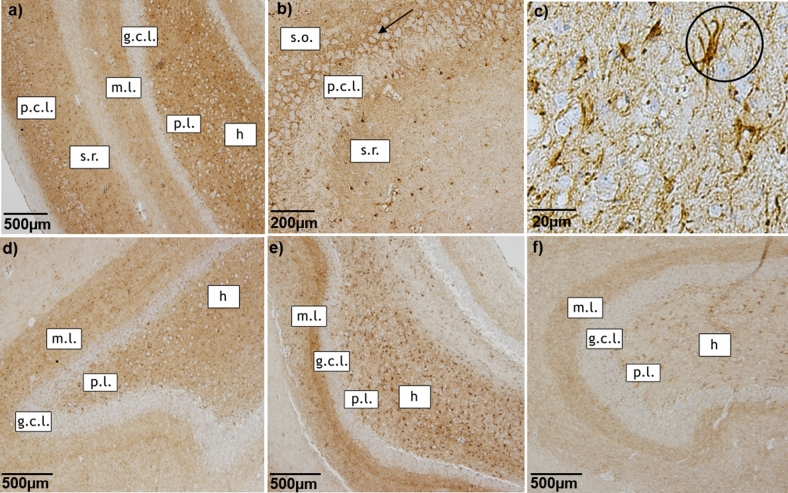
CB1R immunostaining of hippocampus. Representative microphotographs of CB1R immunoreactivity in the hippocampus of a control dog (**a**–**d**), a dog with structural epilepsy (**e**) and a dog with idiopathic epilepsy (**f**). Pyramidal cell layer (p.c.l.) exhibits strong CB1R signal surrounding the unstained soma (**a**, **b**) with intense dot-like CB1R immunoreactivity (arrow in **b**). The dentate gyrus of the hippocampus demonstrates distinct dot-like CB1R immunoreactivity in the molecular layer (m.l.) and hilus (h), whereas granule cell layer (g.c.l.) appears to be devoid of CB1R immunoreactivity (**a**, **d**, **e**, **f**). The circle shows strong CB1R labeling of cells with astrocytic morphology (**c**): p.l.- polymorphic layer; s.o.—stratum oriens, s.r.—stratum radiatum.

CB1R immunoreactivity was observed in all subregions of the hippocampi from all dogs. The expression of CB1R showed major differences between the groups in staining intensity and positive area (Fig. 1).

In the tissue of all dogs, dense CB1R positive staining was visible in pyramidal cell layer in fibers surrounding the pyramidal cells, however the soma itself remained unstained (Fig. 1a,b). Polymorphic and molecular layer of hippocampus also expressed strong CB1R staining (Fig. 1a,b). The strongest immunoreactivity of CB1R was observed in the DG of all animals, particularly in molecular cell layer and hilus (Fig. 1a,d,e,f). Neuronal cell bodies of hilus were not CB1R positive (Fig. 1a,c,d,e,f). However, strong, dense, dot-like CB1R staining was noticed surrounding the soma (Fig. 1). Granule cell layer exhibited no CB1R immunoreactivity (Fig. 1a,d,e,f). Remarkably intense CB1R immunostaining was visible in cells of astrocytic morphology (Fig. 1c).

**Figure 2:**
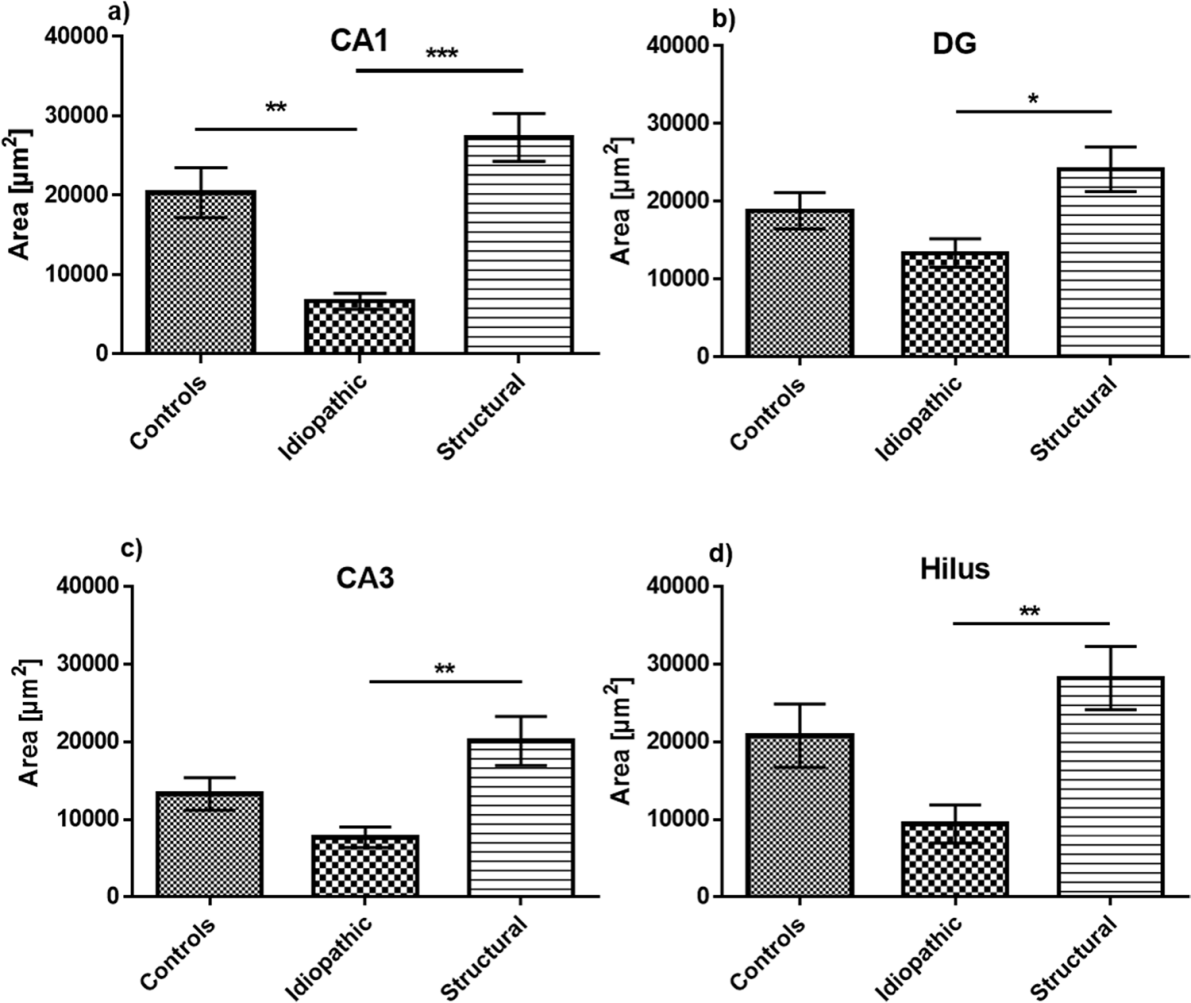
Analysis of the hippocampal CB1R-immunopositive area. In the CA1 region (**a**) the CB1R-immunopositive area was decreased in idiopathic epilepsy in comparison to structural epilepsy and controls. In DG (**b**), CA3 (**c**) and hilus (**d**) the area expressing CB1R in idiopathic epilepsy was significantly smaller in comparison to structural epilepsy, but did not differ significantly to controls; error bars indicate SEM and asterisks in the figures indicate significant differences, *p < 0.05, **p < 0.01, ***p < 0.001; CA: Cornu Ammonis; DG: dentate gyrus.

**Figure 3:**
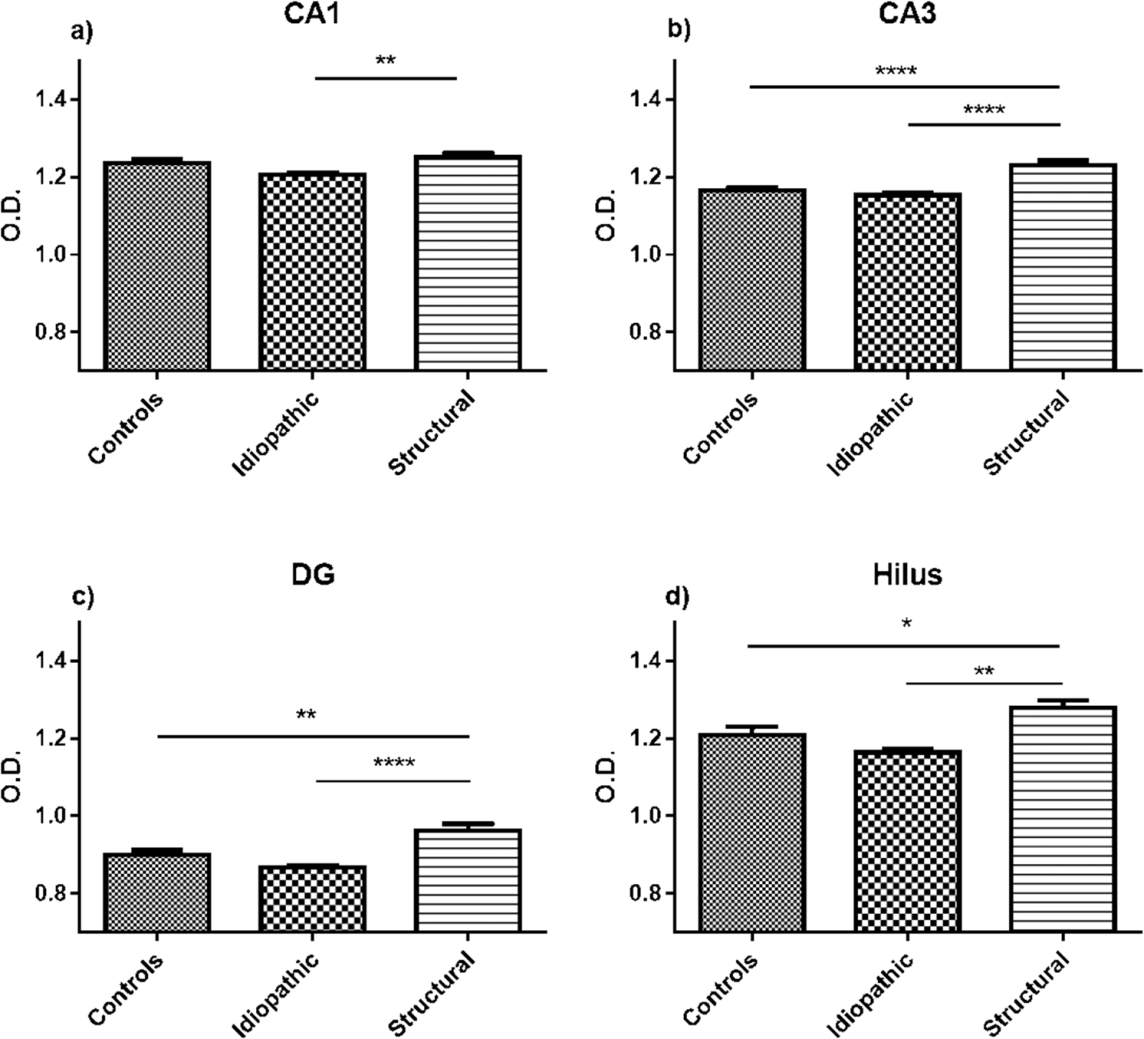
Analysis of intensity of CB1R expression in the hippocampus. In hippocampal sub-regions CA1 (**a**) CA3 (**b**), DG (**c**) and hilus (**d**) the structural epilepsy group showed higher O.D. compared to the idiopathic epilepsy group. Additionally, an increase in O.D. was detected in the structural epilepsy group compared to the control group. Error bars indicate SEM, *p < 0.05, **p < 0.01, ***p < 0.0001; CA: Cornu Ammonis; DG: dentate gyrus; O.D: optical density.

Quantitative analysis of the immunohistochemical staining revealed significant changes in both staining intensity (expressed in O.D.) and in stained area (µm^2^). In the CA1 region of hippocampus, the CB1R-labeled area proved to be smaller in idiopathic than in structural epilepsy patients (*p* < 0.001) and controls (*p* < 0.01) (Fig. 2a). Similar patterns were also observed in other regions of the hippocampus between structural and idiopathic epilepsy groups, however with no difference in respect to the control group. The CB1R-immunopositive area in the DG, CA3 and CA1 was smaller in the tissue from patients with idiopathic epilepsy CB1R as compared to the ones with structural epilepsy (Fig. 2b–d). Similarly, the intensity of CB1R expression in the CA1 area was stronger in dogs with structural than idiopathic epilepsy (*p* < 0.01), with no statistical difference to the controls (Fig. 3a). Interestingly, the intensity of the CB1R expression in the DG, CA3 and hilus was stronger in all patients with structural epilepsy than in tissue of the controls (Fig. 3b–d). In the same regions, hippocampal tissue from dogs with idiopathic epilepsy displayed lower intensity of CB1R expression than the tissue of dogs with structural epilepsy (DG (*p* < 0.0001), CA3 (*p* < 0.0001) and hilus (*p* < 0.01)) (Fig. 3b–d).

**Figure 4:**
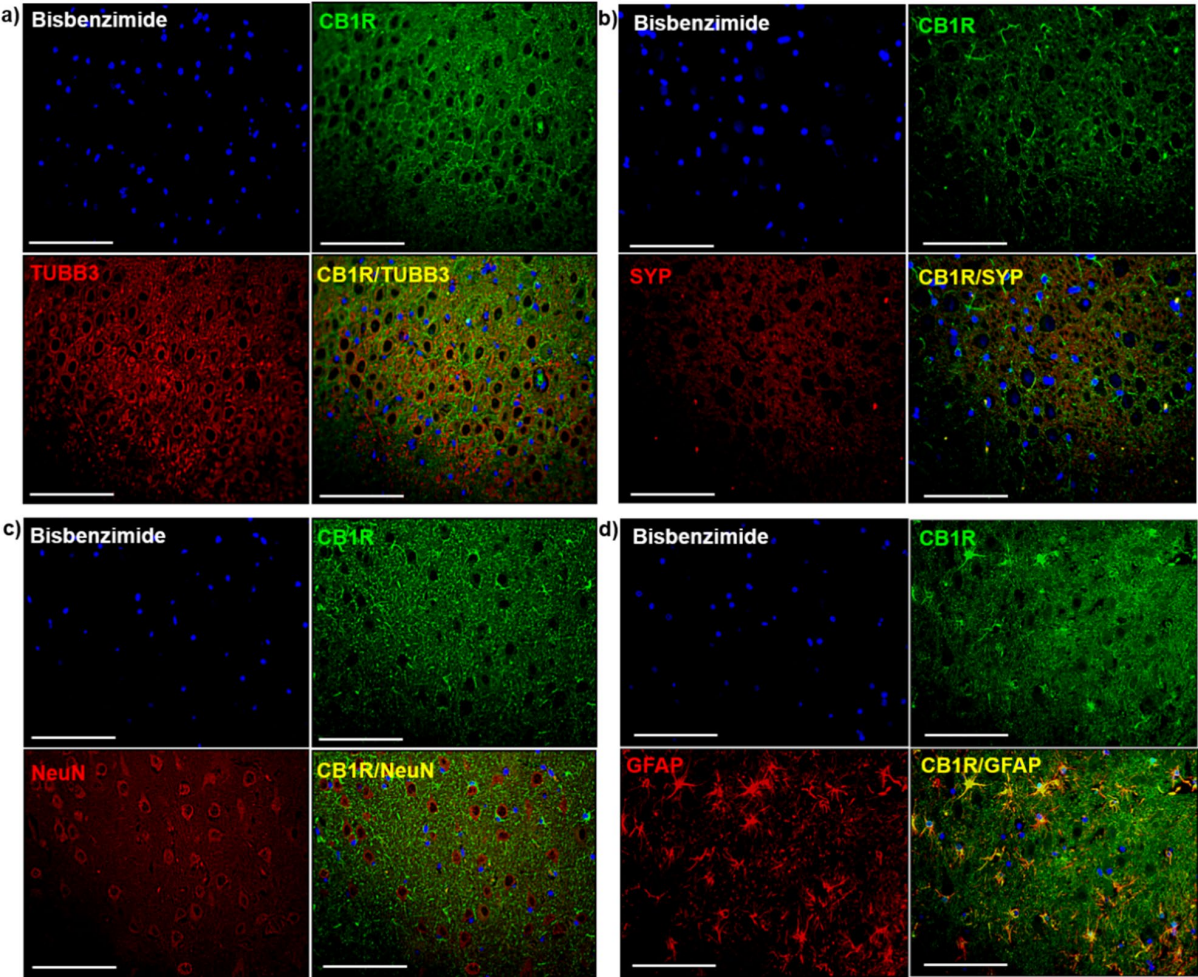
Double immunofluorescence staining of the hippocampus of a control dog. Double immunolabeling did not confirm a co-localization of CB1R with (**a**) TUBB3, (**b**) SYP nor (**c**) NeuN. (**d**) GFAP staining with CB1R showed co-localization of both proteins (CB1RR/GFAP, yellow); (**a**–**d**) Bisbenzimide, nuclear staining (blue); Scale bar = 50 μm (**a**–**d**). CB1R: Cannabinoid receptor type 1; GFAP: glial fibrillary acidic protein; TUBB3: beta-tubulin III; NeuN: neuronal nuclei; SYP: synaptophysin.

Identification of types of CB1R-immunopositive cells was performed by means of immunofluorescent double labeling (Fig. 4, Supplementary Fig. 1). Evaluation of double CB1R staining with TUBB3 (Fig. 4a), a marker for neuronal microtubules and SYP (Fig. 4b), a marker for most neuronal synapses, did not confirm a co-expression of CB1R with any of these neuronal markers. Double staining of CB1R with NeuN was evaluated to assess whether neuronal cell bodies express the receptor, but no co-localization was found (Fig. 4c). However, a marked co-localization of CB1R with GFAP was observed (Fig. 4d), which was previously described in astrocytes of healthy dogs^22^. The co-localization was present in control dogs and patients with epilepsy, in all hippocampal regions, especially emphasized in DG.

**Figure 5:**
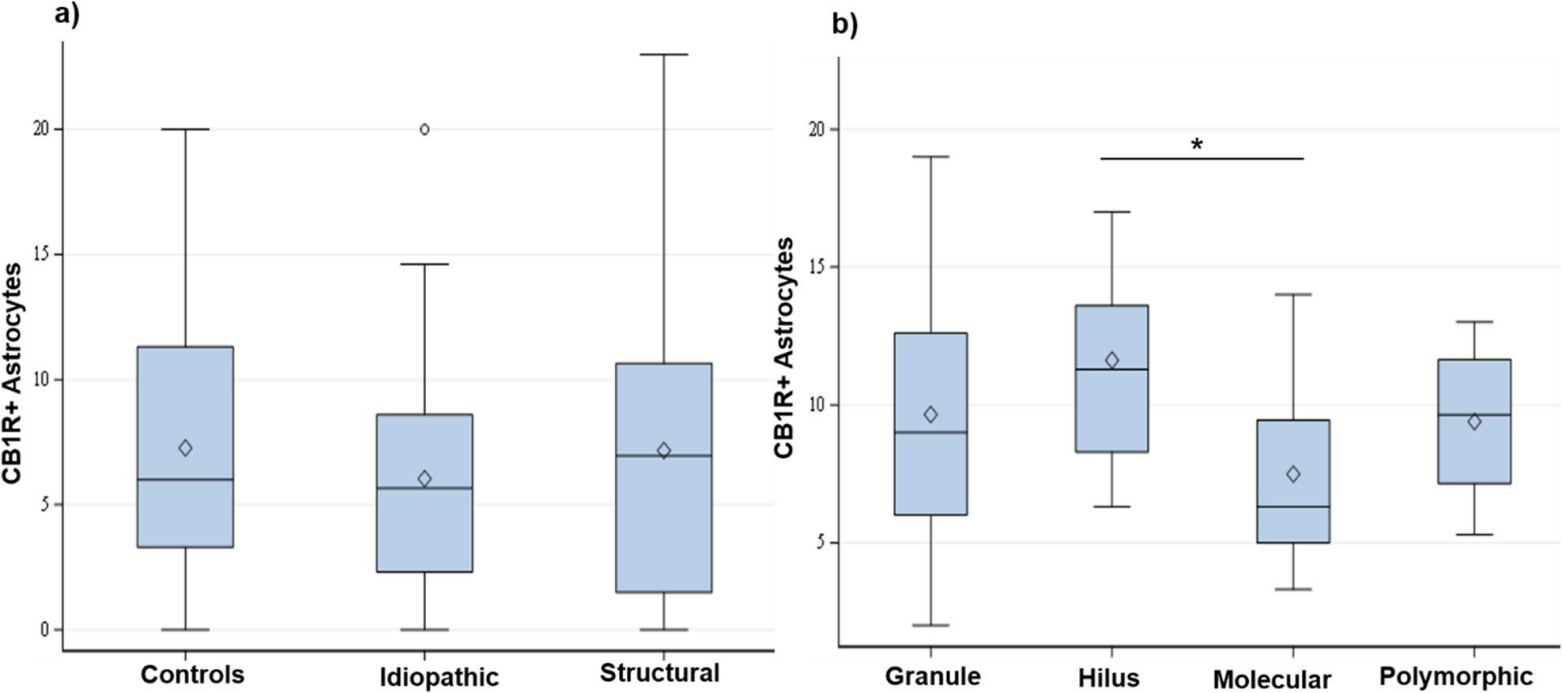
CB1R positive astrocytes quantification. (**a**) Number of CB1R positive astrocytes in different groups and (**b**) different layers calculated for the control group. The cell numbers are expressed per visual field. Boxes represent values from the first to third quartile of CB1R positive astrocytes, inside horizontal lines indicate median values, ◊ represents mean values and vertical lines represent maximum and minimum values; outliers are marked with ○. Asterisk (*) indicate a statistically significant difference (*p* = 0.0044) between the number of CB1R positive astrocytes in the hilus and molecular layer of the dentate gyrus in tissue from control animals. CB1R + : cannabinoid receptor type 1 positive; controls: tissue of control dogs; Idiopathic: tissue of dogs with idiopathic epilepsy; Structural: tissue of dogs with structural epilepsy; Granular: granule cell layer; Molecular: molecular layer; Polymorphic: polymorphic layer.

To quantitatively describe the co-localization of CB1R and GFAP, the number of CB1R positive astrocytes was counted in the DG of control dogs, dogs with idiopathic epilepsy and structural epilepsy by two examiners (DK, JF) with an interrater agreement of 88%. In all groups, the sum of counted CB1R positive astrocytes amounted to more than half of all the astrocytes counted in the DG. There was no statistical difference between the number of CB1R positive astrocytes in control group and idiopathic epilepsy group (*p* = 0.9308), control and structural epilepsy (*p* = 0.1189) nor idiopathic and structural epilepsy group of animals (*p* = 0.1811) (Fig. 5a). Comparison in different layers of the DG revealed no statistically significant differences between numbers of CB1R positive astrocytes in structural epilepsy (*p* = 0.5253) and idiopathic epilepsy (*p* = 0.4118). In contrast, a difference proved to be evident in controls with more CB1R-positive astrocytes in the hilus than in the molecular layer (*p* = 0.0044; Fig. 5b).

## Discussion

To our knowledge, this study was the first to describe disease-associated changes in CB1R expression in hippocampi of canine patients with epilepsy of various etiologies. Reorganization of the receptor was prominent in all animals with epilepsy compared to control dogs without neurologic pathology. Additionally, different patterns of expression modifications were noticeable in tissue from patients with idiopathic epilepsy and structural epilepsy. Furthermore, intense CB1R signal from astrocytes might be of interest for further research of the role of endocannabinoid receptor in this type of cells in dogs.

Qualitative evaluation of immunohistological distribution of CB1R staining in hippocampus of controls and dogs with epilepsy demonstrated strong pointwise immunolabeling of fibers in pyramidal cell layer surrounding unstained neuronal somas and slightly weaker immunoreactivity in hippocampal polymorphic and molecular layer of CA. Similarly, intense CB1R staining was observed in all samples in the dentate gyrus, especially in molecular layer and hilus. These findings are consistent with previously described CB1R expression in healthy dogs^22,23^, as well as in healthy and epileptic experimental animals^14,24,25^. CB1R immunopositive fibers in these sub-regions might be of GABAergic origin, enabling cannabinoid modulation or neurotransmission at perisomatic synapses of pyramidal neurons^26^. The importance of ECS activation on GABAergic neurons was investigated in animal models, in which prolonged febrile seizures led to upregulation of CB1R on this type of neurons^27^.However, in a kainic acid model of TLE, conditional deletion of CB1R from glutamergic and GABAergic neurons led to more deleterious effects in the first case. In the dentate gyrus, strong CB1R immunoreactivity could originate from dense CB1R labeling of mossy fibers^25^. The exact role of the receptor in these fibers is not fully elucidated, although it has been suggested that persistently active cannabinoid receptors turn off the output of mossy fiber associated interneurons in the hippocampal CA3 area^28^. Whether similar patterns are present in canine patients in epilepsy is not described yet, therefore further characterization of CB1R expression in different neuronal population is necessary to better understand the pathology of the disease in this species.

The analysis of immunopositive area and staining intensity revealed an overall increase of CB1R expression in all regions of interest in samples from animals with structural epilepsy, regardless of exact diagnosis. CB1R expression changes were described in human samples of neoplastic, inflammatory and vascular diseases^29–32^, although the influence of given pathologies on CB1R expression often remains controversial. Due to low sample sizes for respective pathologies, we decided to group them under one “structural epilepsy” group. Variances of the signal intensity and staining area for this group seemed to be similar as in the other groups; therefore, we assumed the effect of the specific pathology is less relevant for CB1R expression than the effect of seizure activity.

Seizures themselves can increase the density of CB1R in the pyramidal cell layer^14,18,20^. One of the reasons of increased receptor expression in this sub-region could be sprouting of new fibers of interneurons^20^. One should also account for compensatory mechanisms executed by the ECS resulting in higher production of CB1R in certain subpopulations of excitatory cells in the hippocampus^33^. Both mechanisms might be involved in increased CB1R expression in dogs with structural epilepsy. However, there are contradictory views on how CB1R expression in certain regions of the hippocampus might influence the course of the disease and semiology of seizures. Endocannabinoids could act on receptors at excitatory synapses and suppress seizures by inhibiting glutamate release^34^; conversely, endocannabinoid signaling could promote seizures by inhibiting GABA release at inhibitory synapses^35^. It is likely that this dichotomous behavior of CB1R serves ECS for “fine-tuning” of synapses during development of epilepsy and seizures^36^.

In contrast to brain samples from animals with structural epilepsy, immunohistochemistry of tissue from patients suffering from idiopathic epilepsy revealed a general decrease of CB1R expression. Differences in the expression of CB1R have been also detected in experimental rodent models of temporal lobe epilepsy (TLE) suggesting an influence of epileptogenesis on receptor biosynthesis^14,37,38^. Downregulation of CB1R in hippocampus was also reported in human TLE^17^. To what extent canine epilepsy might be directly compared to human TLE remains unclear^39^, however, hippocampal atrophy, one of hallmarks of TLE in human patients, is associated with epileptic activity at least in some dogs with idiopathic epilepsy^40,41^. Therefore, downregulation of CB1R expression might be connected to the shrinkage of hippocampal formation progressing in the course of the disease. Unfortunately, due to quality and quantity of the archived tissue, an exact analysis of hippocampal atrophy was not possible. It is however conceivable that a decrease in the CB1R expression may contribute to seizure activity, as conditional knock-out of CB1R led to a lower seizure threshold in a kainic acid mouse model^42^, while complete knock-out of genes for cannabinoid receptors provoked spontaneous seizures in a mouse model^43^.

It should be also considered that an increase in intensity only indicates an upregulation of CB1R in cells that also express the receptor at control conditions, whereas an increase in area indicates an induction in cells that normally do not express the receptor at levels above a detection threshold. The observed increase of intensity in the structural epilepsy group and decrease of stained area in the idiopathic group indicate difference in an expression pattern on the cellular level.

CB1R presence is described on axon terminals, interneuron fibers, on synapses and perisomatically^44^. To assess which type of cells express CB1R in dogs, double immunofluorescence staining with markers for neurons, astrocytes, axonal microtubules and synaptic vesicles was performed. CB1R presence was confirmed only in astrocytes, leading to the assumption that detection of CB1R on neuronal body, microtubules and synaptic vesicles in hippocampus is not feasible in dogs using the described method. It is possible that the affinity of used antibodies is influenced differently through the membrane of various cell types or tissue processing modifies epitope presentation on some of the brain cells. Since studies characterizing synapsin expression in canine tissue are scarce^45^, it would be advantageous to describe it in detail in canine tissue of patients without and with epilepsy. Unfortunately, due to scarcity of pathological material, it was not possible to perform it for our study.

Among astrocytes, more than half of the population expressed CB1R, regardless of experimental group. Additionally, astrocytic staining was very intense in all samples. This findings is in apparent contrast to other studies reporting low levels of CB1R expression in astrocytes^46^. Considering their possible involvement in epilepsy and epileptogenesis^47–49^, the high number of astrocytes expressing CB1R in DG in dogs presents an interesting starting point for further evaluation. Activation of astrocytic CB1R leads to an increase of intracellular Ca^2+^ and consequent modulation of the synthesis of proteins involved in neuroplasticity, usually resulting in pronounced long-tern depression^50^, which could be one of possible mechanisms of anti-seizure effect of cannabinoids. The observed reorganization on CB1R positive astrocytes between sub-regions of the dentate gyrus in dogs with epilepsy as compared to control animals might be a consequence of alterations in the communication between neurons and astrocytes, attracting the latter to the molecular layer filled with terminals of axons from the entorhinal cortex. Higher numbers of CB1R positive astrocytes in the dentate gyrus might also be related to increased neurogenesis associated with epileptogenesis. Activation of CB1R is known to increase proliferation of neural precursor cells generally^51^ and their differentiation into astrocytic lineage specifically^52^. Considering the high expression of CB1R in canine astrocytes, this species might offer a suitable model for further research of a possible involvement of the astrocytic ECS in mechanisms of epilepsy and epileptogenesis.

It would be also advantageous to validate those results using another methodology—although studies in animal preclinical models indicate that CB1R availability measured by means of positron emission tomography (PET) and IHC were consistent in telencephalon, in other brain parts PET indicated higher activation than IHC data^53^.

## Conclusion

CB1R in canine hippocampus confirmed a differential expression in patients with epilepsy as compared to animals without neurological pathology. The expression of the receptor was increased in tissue from dogs with structural epilepsy and decreased in canine patients with idiopathic epilepsy. The distinct disease-associated CB1R regulation is an important new aspect in the context of molecular epilepsy-associated alterations, which might be of interest for future development of novel treatment approaches for dogs with epilepsy. The functional relevance of the high astrocytic expression rates of CB1R in tissue from canine patients should be addressed by experimental follow-up studies with a cell-type specific regulation of CB1R in astrocytes.

## Materials and methods

### Study design and animals

Brain tissue of 19 dogs used for staining was collected from the archive of the Department of Pathology, University of Veterinary Medicine Hannover. Sections were selected to match the level of the hippocampus.

For this study, brain samples were grouped according to diagnosis (Table 1, Supplementary Table 1): control (n = 7; age: range 2–120 months, mean ± SEM 36.78 ± 12.32), idiopathic epilepsy (n = 5; age: range 5–96 months, mean ± SEM 30.2 ± 16.7) and structural epilepsy (n = 7, age range 3–132 months, mean ± SEM 73.71 ± 20.53). The tissue of all dogs was evaluated and the diagnosis was established by a board-certified pathologist. In dogs with idiopathic epilepsy, no evident histopathological changes in brain parenchyma were noted. Samples of dogs with structural epilepsy had noticeable changes in parenchyma, presumably causing the clinically observed seizures: one dog with hydrocephalus, one with cerebellar infarct, three dogs with brain tumor, one dog with encephalitis and one with periventricular vacuolization. All dogs with seizures were diagnosed with epilepsy during clinical workup (Table 1). Brain tissue was collected after patients’ exitus or euthanasia in the course of clinical treatment. Time between tissue collection and further pathomorphological processing did not exceed 3 days. Tissue collection and further study were performed according to the German Animal Welfare law, as well as Universities ethical regulations. Control brain tissue was selected from the above-mentioned archive and came from animals without any clinical or neuropathological evidence of CNS disease (Supplementary Table 1). The study was accepted by the PhD committee of University of Veterinary Medicine Hannover Foundation. No animal was euthanized for the purpose of this study specifically. All the owners of canine patients included in the study signed an informed consent regarding the use of the biological material in research. Samples from the experimental control group originated from healthy beagles, euthanized for another study^54^ with the animal experiment number 33.9-42,502-05-13A346. Since this study did not include any animal experiment, only animal tissue, no specific ethical committee approval nor the ARRIVE guidelines apply.

**Table 1:** Patient information for dogs with epilepsy included in the study. Clinically relevant information regarding canine patients with epilepsy.

Patient ID	Age (months)	Sex	Breed	Type of epilepsy	Type of seizure	Brain pathology	First seizure event before clinical presentation (months)	Onset of Seizures/Cluster/status epilepticus (SE) before euthanasia/ death	Seizure frequency	Days between death and tissue processing
1	5	m/–	Pug	Idiopathic, subtype: unknown cause	Cluster	–	2.5	> 1 day	1-6 ×/day	3
2	23	m/–	West Highland White Terrier	Idiopathic, subtype: unknown cause	Cluster	–	18	> 1 day	Cluster every day	1
3	48	f/–	German Shepherd	Idiopathic, subtype: unknown cause	Cluster	Ectopic Purkinje cells in the cerebellum (without clinical relevance)	1	Unknown	Several in a day	1
4	96	m/c	Border Terrier	Idiopathic, subtype: unknown cause	Cluster	–	72	> 1 day	2-3 ×/week	2
5	18	f/c	Mixed	Idiopathic, subtype: unknown cause	Cluster	–	12	Unknown	Unknown	2
6	66	m/–	Pug	Idiopathic, subtype: unknown cause	Cluster	Ventriculomegaly	12	2 days	1 ×/month	< 1
7	157	m/–	Dachshund	Idiopathic, subtype: unknown cause	Cluster	Low-grade multifocal vacuolisation of the white matter	132	3 days	1 ×/month	< 1
8	2.5	m/–	Cocker Spaniel	Idiopathic, subtype: unknown cause	Status epilepticus (SE)	within the Ammon's horn: segmental distribution of basophile shrivelled neurons (artifacts or due to hypoxia)	2	> 5 days	Unknown	0
9	132	m/–	Cocker Spaniel	Structural epilepsy; epilepsy caused by identified cerebral pathology	Single seizures	Vacuolisation in the frontal white matter	6	Unknown	2 ×/month	0
10	38	m/–	Unknown	Structural epilepsy; epilepsy caused by identified cerebral pathology	Cluster	Hydrocephalus	1	> 1 day	2-3 ×/day	0
11	60	f/–	Pug	Structural	Cluster	Meningoencephalitis: “pug dog encephalitis”, necrotizing encephalitis	1	> 16 h	1-2 ×/month	2
12	66	f/c	Poodle	Structural	Cluster	Granulomatous meningoencephalitis (infectious origin arguable)	1	4 days	Unknown	1
13	132	f/c	Spanish Waterdog	Structural epilepsy; epilepsy caused by identified cerebral pathology	Cluster	Encephalomalacia (cerebral infarct feasible)	Unknown	Unknown	Unknown	3
14	132	m/–	Boxer	Structural epilepsy; epilepsy caused by identified cerebral pathology	Cluster	Glioblastoma	Unknown	2 days	3 × before death	1
15	30	m/–	French Buldog	Structural epilepsy; epilepsy caused by identified cerebral pathology	Cluster	Low-grade focal haemorrhage in the 4th ventricle, low-grade internal hydrocephalus with atrophy of the white matter, moderate multifocal to diffuse periventricular vacuolisation in the hippocampus	18	Unknown	6-7 ×/day	3
16	140	f/–	Mixed	Structural epilepsy; epilepsy caused by identified cerebral pathology	Cluster	Toxoplasmosis, leucoencephalomalacia (non infectious origin feasible, e.g. hypoxic); multifocal neuronal necrosis in the cerebral cortex and hippocampus, cerebellar atrophy	18	> 4 h	4 ×/day	1
17	36	m/c	Unknown	Structural epilepsy; epilepsy caused by identified cerebral pathology	SE	Fibroplastic meningioma, purulent encephalitis and malacia; reactive encephalitis caused by the tumor	9	> 1 h	1 ×/month	0
18	36	f/c	Unknown	Structural epilepsy; epilepsy caused by identified cerebral pathology	SE	Meningioma in the olfactory bulb, purulent necrotic inflammation (bacterial, reactive, caused by the tumor), satellitosis in the cerebral cortex	36	> 3 days	Unknown	0
19	79	f/–	Maltese	Structural epilepsy; epilepsy caused by identified cerebral pathology	SE	Moderate to high-grade multifocal granulomatous meningoencephalitis	79	7 days	Unknown	1
20	120	f/–	Vizsla	Structural epilepsy; epilepsy caused by identified cerebral pathology	SE	Meningoencephalitis	119	Unknown	Unknown	1

m/– male, not castrated; f/– female, not castrated; *m/c* male, castrated; *f/c* emale, castrated; *SE* status epilepticus.

### Tissue preparation

Brain tissue was prepared for histological evaluation immediately after necropsy. The tissue was fixed in non-buffered formalin (10%), embedded in paraffin and cut into serial sections of 3 µm thickness. For histopathological analysis, sections were mounted onto SuperFrost-Plus microscope slides (Menzel Gläser, Braunschweig, Germany) and stained with hematoxylin and eosin (HE, Sigma-Aldrich, CID 442,514, Merck KGaA, Darmstadt, Germany). Transversal cuts of brain tissue at the level of hippocampus were mounted onto microscope slides and processed for further immunohistochemistry.

### Antibodies

Immunohistochemistry (IHC) and immunofluorescence (IF) of the selected tissue was performed using a polyclonal antibody against CB1R (Abcam Cat# ab23703, RRID: AB_447623, dilution 1:200 IHC, 1:20 IF), the immunogen corresponding to C terminal amino acids 461 ± 472 of human cannabinoid receptor. For double immunofluorescent labeling, the following monoclonal antibodies were additionally used: anti-glial fibrillary acidic protein (GFAP, Sigma-Aldrich Cat# G-A-5, RRID: AB_2314539, dilution 1:500), anti-beta-tubulin III (TUBB3, Sigma-Aldrich Cat# T8660, RRID: AB_477590, dilution 1:200), anti-neuronal nuclei (NeuN, Millipore Cat# MAB377, RRID: AB_2298772; dilution 1:800), anti-synaptophysin (SYP, Dako Cat# M0776, RRID: AB_2199013; dilution 1:250).

### Immunohistochemistry

In order to evaluate CB1R expression, brain tissue was immunohistochemically stained using previously established avidin–biotin-peroxidase complex (ABC) method^22,55^. Briefly, after deparaffinization and rehydration through a series of xylene and gradient ethanol to water, sections were treated with 0.5% H_2_O_2_ in methanol to block endogenous peroxidase. For antigen retrieval, sections were transferred into sodium citrate buffer (pH 6.0–6.5) and heated for 20 min in the microwave at 800 W. After 20 min incubation with 20% solution of normal goat serum diluted in phosphate-buffered saline (PBS) to block unspecific protein binding, tissue was subsequently incubated with the CB1R primary antibody overnight at 4 °C. A slide cut at the level of cerebellum was used as a positive control^22^. Positive and negative controls were treated using the same protocol. In the negative control, primary antibody was substituted with rabbit serum (R4505; Sigma Aldrich, Taufkirchen, Germany; dilution 1:3000). Biotin-labeled goat-anti-rabbit IgG (Vector Laboratories Cat# BA-1000, RRID: AB_2313606; dilution 1:200), was added as secondary antibody and incubated for 45 min at room temperature. Subsequently, ABC was applied to amplify the signal (Vector Laboratories Cat# PK-6100, RRID: AB_2336819). The staining was developed by a color reaction of 3.3'-diaminobenzidine tetrahydrochloride (0.05% solution, DAB, Cat # D 3939, Sigma Aldrich, Taufkirchen, Germany) with H_2_O_2_ (0.03%, pH 7.2). In the last steps, the slides were counterstained with Mayer’s hematoxylin, dehydrated and cleared in acetic acid-n-butyl ester (EBE, Roth, Karlsruhe, Germany), and mounted using Roti-Histokit (Roth, Cat # 6638, Karlsruhe, Germany). Histological evaluation of the receptor’s expression was performed using a bright field microscope (Olympus BH2, Olympus Optical CO., Tokyo, Japan) with single chip charge-coupled device (CCD) color camera (Axiocam; Zeiss, Göttingen, Germany) and processed with image capture interface software (Axiocam MR Interface Rev.A; Zeiss, Göttingen, Germany).

### Double immunofluorescence

Brain slides were analyzed in the context of co-localization of CB1R and GFAP, NeuN, TUBB3 and SYP using a double immunofluorescence method as previously described^22,55^. All slides were incubated with the respective primary antibodies for 90 min. Alexa Fluor 555-labeled goat anti-mouse (Cat # A32727, Life Technologies, dilution 1:200) and Alexa Fluor 488-labeled goat anti-rabbit (Cat # A32731, Life Technologies; dilution 1:200) secondary antibodies were applied for 45 min at room temperature to visualize the respective antigens. Nuclear counterstaining was performed with 0.01% bisbenzimide (H33258, Sigma Aldrich, Taufkirchen, Germany) and slides were mounted with Dako Fluorescent Mounting medium (DakoCytomation, Cat # S3023, Hamburg, Germany). The double labeling CB1R/GFAP, CB1R/NeuN, CB1R/TUBB3 and CB1R/SYP was visualized using an inverted fluorescence microscope (BZ-9000E, Keyence GmbH, Neu-Isenburg, Germany) and examined through the BZ-II Analyzer software. Images of each sample were captured using fixed microscope settings under which the negative control sections showed no signal.

### Qualitative and quantitative tissue analysis

In each tissue sample, the evaluation of immunohistochemistry and immunofluorescence was performed at the level of the hippocampus (canine brain transections http://vanat.cvm.umn.edu/brainsect/). Quantitative evaluation of immunohistochemical staining was performed in dentate gyrus (DG), Cornu Ammonis (CA)1 and CA3. Due to limited availability of archived tissue material double immunofluorescence staining could only be performed in DG.

Quantitative analysis of immunostaining of CB1R was performed using ImageJ software (U.S. National Institutes of Health, Bethesda, [https://imagej.nih.gov/ij/] ImageJ, RRID:SCR_003070). Firstly, RGB images were converted in 8-bit greyscale for quantitative analysis. Images were then both spatially calibrated with 25 µm calibration grid slide (MBF Bioscience, Vermont, USA) and grey values were calibrated for optical density (O.D.). The latter was performed by means of ImageJ optical density calibration protocol based on a Tiff image file of a Kodak No. 3 Calibrated Step Tablet scanned with an Epson Expression 1680 Professional scanner. The tablet consists of 21 steps with a density range of 0.05–3.05 O.D., which corresponded to specific greyscale values after the calibration. In each hippocampus region, frames were randomly chosen, using 3–5 regions of interest (66,280 µm^2^ each), which resulted in 9–15 pictures per animal. Grey value of each image was thresholded using ImageJ default mask, a variation of the IsoData algorithm^56^, then quantified for the total area expressed in µm^2^ and for the mean O.D values, representatives of the CB1R expression extension and intensity. For each of these steps, the investigator was blinded as for group affiliation of a given sample.

Double immunofluorescence-stained tissue was also evaluated quantitatively using ImageJ. The cell-counter tool of the software was applied on acquired images of different DG layers: polymorphic, molecular, granule cell layer and hilus. CB1R/GFAP double positive cells were counted on the computer screen by two investigators unaware of the sample identity in up to five counting frames for each layer. The number of CB1R positive astrocytes was compared between the groups in the entire dentate gyrus as well as in its respective layers.

### Statistical analysis

Statistical analysis was performed using the SAS^®^ program package, version 9.2 (SAS Institute, Cary, NC, USA) and GraphPad Prism 6 (GraphPad Prism, RRID:SCR_002798) for repeated measures analysis of variance (ANOVA), the pairwise Tukey test and the Kruskal–Wallis test. The assumption of normal distribution of quantitative parameters was examined using the Kolmogorov–Smirnov test and visual assessment of QQ plots of model residuals. Inter-observer agreement for quantitative evaluation of double immunofluorescence staining was tested using interclass correlation test (ICC). All analyses were considered statistically significant for *p*-value < 0.05.

## Supplementary Information

Supplementary Information.

## Data Availability

The authors confirm that the data supporting the findings of this study are available within the article. Brain slides are stored in the archive of the Department of Pathology, University of Veterinary Medicine Hannover Foundation.
