# Supplementary material for: Hippocampal expression of the cannabinoid receptor type 1 in canine epilepsy
Source: Sci Rep. 2023 Feb 23;13:3138. doi: 10.1038/s41598-023-29868-3 (PMC9950490; doi:10.1038/s41598-023-29868-3)
Supplement: Supplementary file 1 — Supplementary Information. [file 41598_2023_29868_MOESM1_ESM.docx]

**Supplementary figure 1. Imaging site for fluorescent pictures.**


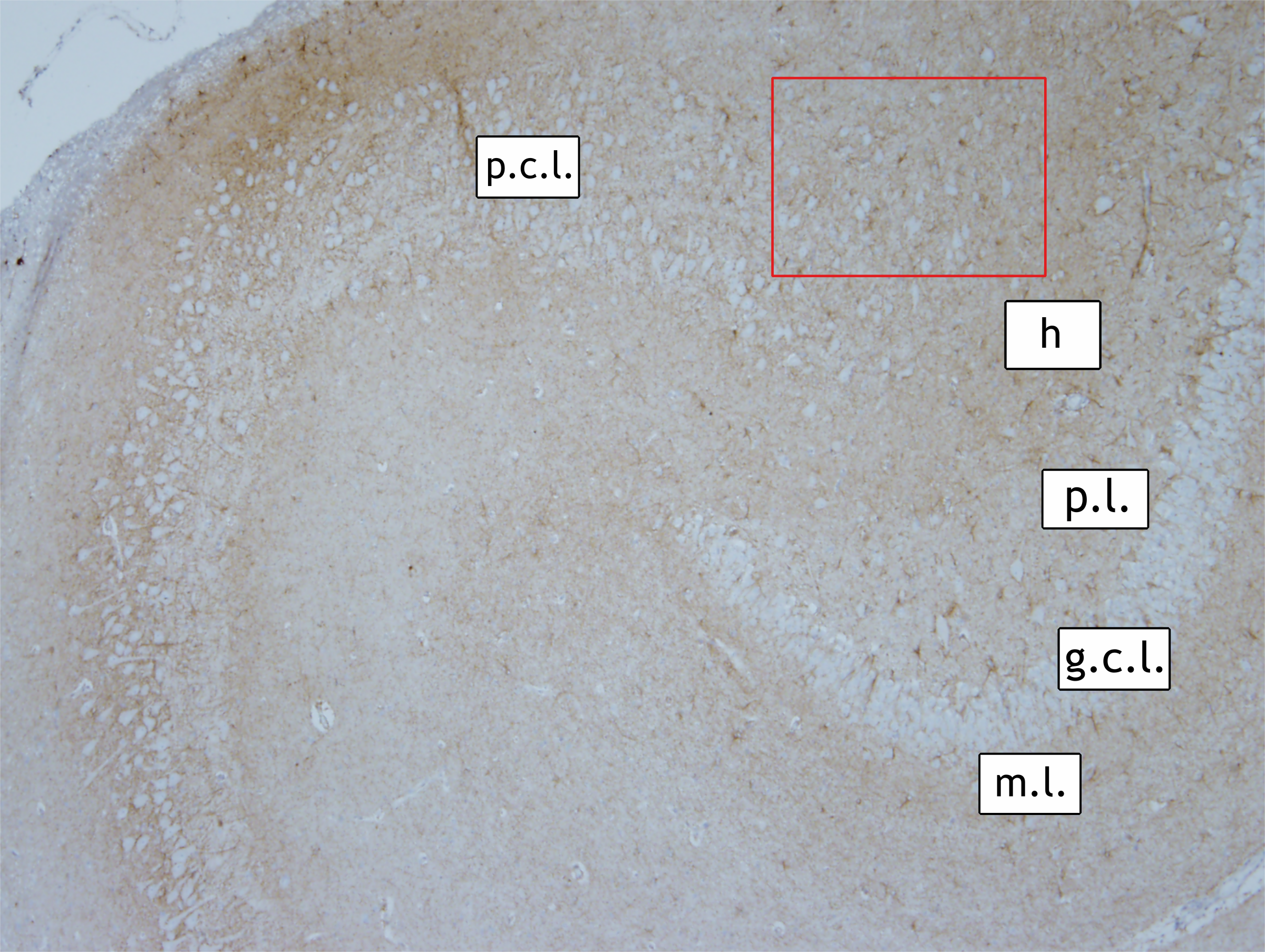
Red rectangle indicates where fluorescent pictures presented in the Figure 4 were captured. Depending on the slice, the pictures were taken either in the dorsal or in the ventral portion of the hippocampus. m.l. - molecular layer, g.c.l. – granular cell layer, p.l. – polymorphic layer, h – hilus, p.c.l. – pyramidal cell layer

**Supplementary table 1.** **Patient information for control dogs included in the study.**

| **Patient ID** | **Age (months)** | **Sex** | **Breed** | **Control type** | **General pathology** | **Days between death and tissue processing** |
| --- | --- | --- | --- | --- | --- | --- |
| 21 | 2 | m/- | Pinscher | Patient | Right-sided aortic arch; ventricular septal defect | 1 |
| 22 | 5 | f/- | Labrador mixed breed | Patient | Multiple hematomas and hemorrhages; coagulation dysfunction, suspicion of intoxication | 1 |
| 23 | 18 | m/c | Mixed breed | Patient | Multiple hemorrhages; dermatitis, folliculitis, furunculosis, hyperkeratosis (Demodex canis); nephritis punctata | 3 |
| 24 | 23 | f/c | Mixed breed | Patient | Multifocal mineralization of dura mater (not clinically relevant); foreign bodies in stomach, gastritis; hyperplasia of lymph nodes and spleen | 1 |
| 25 | 32 | m/c | Great Dane | Patient | Right ventricle dilatation, myocardium fibrosis; lung hyperemia and alveolar edema | unknown |
| 26 | 36 | m/- | Kangal | Patient | Lung hyperemia and alveolar edema | 1 |
| 27 | 36 | m/- | German Spaniel | Patient | Plasmocytoma (humerus) | unknown |
| 28 | 38 | m/- | Mixed breed | Patient | unknown | unknown |
| 29 | 48 | f/c | Bernese Mountain Dog mixed breed | Patient | Lung hyperemia and alveolar edema | 1 |
| 30 | 60 | m/- | German Shepherd | Patient | Acute dilatation of right atrium; multiple organ congestion; pulmonary alveolar edema and emphysema; multifocal hemorrhages in cutaneous and subcutaneous layers | 1 |
| 31 | 66 | f/- | German Shepherd | Patient | Multiple organ congestion, erythrophagocytosis | 1 |
| 32 | 96 | f/- | Pekingese | Patient | Pyelonephritis, sepsis | unknown |
| 33 | 108 | f/c | Kuvasz | Patient | Gonarthritis purulenta, phlegmona in the wound area | 0 |
| 34 | 120 | f/- | American Cocker Spaniel | Patient | Moderate hepatonecrosis, purulent inflammation; adenocarcinoma (mammary gland), leiomyoma in myometrium and hyperplasia of endometrium | 1 |
| 35 | 132 | m/- | Collie | Patient | Carcinoma with angiosis carcinomatosa (thyroid gland), Leydig cell tumour (both testicles), nodular spleen hyperplasia, follicular gastritis | 2 |
| 36 | 132 | f/- | Mixed breed | Patient | Hemangiosarcoma (heart - right auricle, spleen), leiomyoma (uterus), cystadenoma (mammary gland), multiple metastases in lungs; high degree of pancreas atrophy | 2 |
| 37 | 140 | m/c | German Shepherd | Patient | Splenic and gastric torsion; pulmonary alveolar edema and emphysema | 3 |
| 38 | 180 | m/- | Standard Long-haired Dachshund | Patient | Peridontitis, rhinitis, hepatitis, hepatolipidosis, focal pneumonia, endocardiosis | 1 |
| 39 | 12 | m/- | Beagle | Experimental | Infection with Angiostrongylus vasorum | unknown |
| 40 | 12 | w/- | Beagle | Experimental | Infection with Angiostrongylus vasorum | unknown |
| 41 | 12 | w/- | Beagle | Experimental | Infection with Angiostrongylus vasorum | unknown |
| 42 | 12 | w/- | Beagle | Experimental | Infection with Angiostrongylus vasorum | unknown |
| 43 | 12 | m/- | Beagle | Experimental | Infection with Angiostrongylus vasorum | unknown |
| 44 | 4 | m/- | Beagle | Experimental | Infection with Angiostrongylus vasorum | unknown |
| 45 | 4 | w/- | Beagle | Experimental | Infection with Toxocara canis and Uncinaria stenocephala, intermittent diarrhea | unknown |
| 46 | 4 | m/- | Beagle | Experimental | Infection with Toxocara canis and Uncinaria stenocephala, intermittent diarrhea | unknown |
| 47 | 4 | w/- | Beagle | Experimental | Infection with Toxocara canis and Uncinaria stenocephala, intermittent diarrhea | unknown |
| 48 | 4 | w/- | Beagle | Experimental | Infection with Toxocara canis and Uncinaria stenocephala, intermittent diarrhea | unknown |
| 49 | 4 | m/- | Beagle | Experimental | Infection with Toxocara canis and Uncinaria stenocephala, intermittent diarrhea | unknown |
| 50 | 4 | m/- | Beagle | Experimental | Infection with Toxocara canis and Uncinaria stenocephala, intermittent diarrhea | unknown |

Clinically relevant information regarding canine patients without CNS disease and experimental controls.

m/- - male, not castrated; f/- - female, not castrated; m/c – male, castrated; f/c – female, castrated
